# Robust Detection of Small Moving Objects Against Real-World Complex Dynamic Natural Environments: Drosophila-Inspired Visual Neural Pathway Modeling

**DOI:** 10.3390/biomimetics11050333

**Published:** 2026-05-09

**Authors:** Sheng Zhang, Ke Li, Zhonghua Luo

**Affiliations:** 1 School of Information and Artificial Intelligence, Wuhu Vocational Technical University, Wuhu 241006, China; 2 College of Information Science and Engineering, Hohai University, Changzhou 213200, China; 3 School of Mechanical and Electrical Engineering, Nanchang Institute of Technology, Nanchang 330044, China; kelitnit@gmail.com (K.L.); zhonghualuonit@gmail.com (Z.L.)

**Keywords:** Drosophila-inspired visual models, LC11 neurons, mushroom body, small moving objects, real-world complex dynamics natural environments

## Abstract

Currently, small moving object detection remains a highly challenging problem, primarily attributable to four critical factors: limited pixel coverage, blurred texture features, indistinguishability from small-object-like background features (i.e., false positives), and vulnerability to environmental noise interference. The remarkable sensitivity of the Drosophila visual system to small moving objects, which originates from a specialized type of neuron known as “lobula columnar 11” (LC11), has provided inspiration for addressing this challenge. Current bio-inspired visual models have achieved certain advances. However, detection performance against real-world complex dynamic natural environments still requires further improvement. To address the challenge of limited detection accuracy for small moving objects against real-world complex dynamic natural environments, this paper proposes a Motion Small Object Detection (MSOD) model inspired by the Drosophila Vision Small Object Motion Sensitivity (DVSOMS) mechanism, namely DVSOMS-MSOD. The model consists of four stages: The first stage is preliminary processing of visual stimuli, where visual stimuli are perceived, converted to grayscale, and blurred. The second stage is the motion neural pathway, where visual signals are first decomposed into parallel ON and OFF neural pathway signals; then, the neural feedback mechanism is incorporated between the medulla and lobula complex, and the complete Hassenstein–Reichardt correlator (HRC) is integrated into the lobula complex; finally, the LC11 neuron is utilized to detect small moving objects and extract their location information. The third stage is the contrast neural pathway, where visual signals are first processed by the central and surrounding local neighborhoods, then local contrast information is calculated. The fourth stage is the integration of motion and contrast neural pathways, where the mushroom body generates motion trajectories using the location information of small moving objects, and subsequently generates contrast trajectories using the local contrast information and motion trajectories to more finely detect small moving objects. Under real-world complex dynamic natural environment datasets, compared with conventional machine learning methods for moving object detection, the proposed model achieved improvements of 77.82% and 78.70% in detection performance and output quality, respectively, while reducing running time by 10.60%. Compared with bio-inspired visual models for small moving object detection, the proposed model achieved improvements of 28.24% and 43.15% in detection accuracy and detection performance, respectively, but the running time increased by 43.40%. The proposed model demonstrates certain advantages in detection performance, output quality, and detection accuracy; however, its real-time performance still warrants further optimization.

## 1. Introduction

Moving object detection is a core technology in computer vision, widely applied in intelligent surveillance [1], autonomous driving [2], and unmanned aerial vehicle (UAV) tracking [3]. However, detecting small moving objects remains highly challenging due to their limited pixel areas, severe texture detail loss, and susceptibility to background clutter and noise [4]. While previous studies have predominantly relied on machine learning approaches, the Drosophila visual system has been largely overlooked despite its demonstrated high sensitivity to small moving objects [5]. As a model organism, Drosophila possesses an efficient and compact visual neural system essential for survival behaviors such as prey chasing and mate finding [6,7]. Several motion-sensitive neurons have been identified, including the lobula plate tangential cell (LPTC) [8,9], direction selective neuron (DSN) [10], lobula giant movement detector 1/2 (LGMD1/2) [11], lobula plate—lobula columnar 1/2 (LPLC1/2) [12,13], small target motion detector (STMD) [14,15], and LC11 [16]. LPTC responds to wide-field and local salient motion; DSN exhibits directional preference; LGMD1/2 and LPLC1/2 respond vigorously to looming objects; STMD and LC11 show exquisite selectivity for dim, small moving objects, with STMD displaying direction selectivity while LC11 does not. Most research has focused on STMD, with limited attention to LC11.

The complete visual neural pathway of Drosophila comprises the retina, optic lobe, and central brain, which play a crucial role in detecting small moving objects [17]. The first-order structure, the retina, contains numerous ommatidia, each consisting of eight photoreceptors, photoreceptor 1–8 (R1–R8); R1–R6 capture luminance information, while R7–R8 capture color information [18,19]. Notably, the retina exhibits significant blurriness [20]. The second-order structure, the optic lobe, consists of the lamina, medulla, and lobula complex. In the lamina, the lamina monopolar cells (LMCs), including the luminance-increase-sensitive L1 and luminance-decrease-sensitive L2, receive signals from R1–R6 [21]. The LMCs also employ lateral inhibition to enhance spatial contrast and motion boundary selectivity [22]. Additionally, local contrast is computed by the amacrine cell (AMC) and tangential 1 (T1) neurons [23,24]. In the medulla, the transmedullary 1–3 (Tm1–Tm3) and medulla intrinsic 1 (Mi1) neurons receive LMC signals, with Tm3 and Mi1 responding to luminance increases and Tm2 and Tm1 to decreases; notably, Mi1 and Tm1 exhibit delayed responses relative to Tm3 and Tm2, respectively [25]. Finally, lobula complex neurons such as LC11 integrate medullary and feedback signals to selectively respond to small moving objects [5,16]. The third-order structure, the central brain (including the central complex and mushroom body), serves as the highest-order processing center, though the integration of motion and contrast information remains incompletely understood. In complex and dynamically varying natural environments, contrast (a measure of luminance discrepancy between objects and backgrounds) provides critical discriminative features for detecting moving objects [26,27], motivating extensive research in computer vision and machine learning on contrast-based detection methods.

Although Drosophila-inspired models based on visual neural circuits have made certain advances in detecting moving wide-field and salient objects [28,29], they remain insufficiently explored for small moving object detection. Meanwhile, such detection is strongly influenced by object size and texture, false positives from the background, and environmental noise, resulting in insufficient detection performance. This paper proposes the DVSOMS-MSOD model that integrates the neural feedback mechanism and the parallel motion-contrast pathway to address these challenges. To sum up, the main contributions of this study are as follows:

(1) An improved temporal band-pass filter is newly proposed to simulate the high luminance change sensitivity of LMC neurons; this achieves more accurate simulation of biological visual luminance perception characteristics.
(2) A neural feedback mechanism is introduced between the medulla and lobula complex, thus suppressing environmental noise interference effectively. Meanwhile, the complete HRC pathway is embedded into the lobula complex; this greatly enhances the ability to perceive and detect small moving objects.
(3) The motion pathway and contrast visual neural pathway are fused at the mushroom body. This design effectively suppresses background false positive interference and realizes precise detection of small moving objects.
(4) A three-layer visual neural circuit computational model of Drosophila is constructed. It adopts a hierarchical perception strategy to achieve robust detection of small moving objects in complex dynamic natural environments, and provides a novel bio-inspired solution for designing a high-performance small moving object detector.

The rest of this paper is organized as follows. An overview of the related works is provided in Section 2. The DVSOMS-MSOD model is introduced in detail in Section 3. Section 4 describes the experiments and performance analysis. Limitations and future research directions are discussed in Section 5. Section 6 concludes this paper.

## 2. Related Works

In recent years, numerous studies have explored Drosophila’s visual system. Notable research directions include neural feedback mechanisms, as well as motion and contrast visual neural pathways, all of which will be discussed below.

### 2.1. Neural Feedback Mechanisms

Neural feedback mechanisms represent a critical information processing strategy in Drosophila’s visual system [30]. The core of this mechanism is the feedback of output signals from higher-order neurons to lower-order neurons, thereby dynamically modulating the response properties of the latter. This mechanism enables insects to suppress environmental noise effectively, significantly enhancing the efficiency and accuracy of visual information processing. For instance, Drosophila relies on this visual feedback pathway to achieve rapid adjustments to flight posture, thus maintaining visual stability and enhancing adaptability in complex and dynamic natural environments [31]. In recent years, researchers have successfully incorporated feedback mechanisms into artificial neural networks, achieving remarkable results in tasks such as image super-resolution [32] and image enhancement [33]. Inspired by Drosophila’s visual system, neural feedback mechanisms have gradually been applied to the fields of small moving object detection and collision detection. The relevant literature reviews are shown in Table 1. Given the effectiveness of neural feedback mechanisms in suppressing environmental noise, this study integrates neural feedback mechanisms into the medulla and lobula complex of the LC11-based bio-inspired visual model.

### 2.2. Drosophila’s Parallel Motion and Contrast Visual Neural Pathways

Visual signals in natural environments exhibit pronounced spatio-temporal variability: temporally, dynamic changes in illumination intensity induce signal fluctuations; spatially, variations in topography and geomorphology give rise to textural heterogeneity. Such variability arises from the coupling of multiple environmental factors, reflecting the inherent complexity and dynamic nature of natural systems. Building upon this foundation, spatio-temporal variability drives dynamic alterations in the relative disparities between figure and ground across spatial structure, luminance distribution, and textural features, ultimately manifesting as continuous variations in figure–ground spatial contrast—the most salient representation of natural visual signals in the spatial dimension. These dynamic changes in figure–ground spatial contrast exert two opposing effects: on the one hand, they induce significant fluctuations in the output responses of bionic models, thereby compromising detection stability; on the other hand, these dynamic properties serve as critical discriminative features for distinguishing real small moving objects from spurious small-object-like background features (i.e., false positives).

Regarding the former issue, early research employed environmental statistical analysis [39] and photoreceptor gain control [40], focusing on the analysis of overall contrast differences in visual signals, yet these approaches exhibited significant limitations in scenarios involving local contrast variations. In recent years, studies in biological vision and bionic computation have demonstrated that the synergistic mechanism between contrast normalization and the parallel motion-contrast pathway offers a bionic approach to addressing this challenge. The contrast normalization mechanism [41] serves as a crucial pre-processing stage for signal processing in biological visual systems. Its essence lies in an adaptive gain control mechanism that precedes motion information processing and is primarily implemented through the signal integration function of medulla inter-neurons in the optic lobe. Specifically, while receiving signals from foreground objects, these neurons simultaneously receive feedback from adjacent background regions. Through spatial integration of background signals, they modulate the intensity of foreground signals, thereby achieving adaptive gain control. Concurrently, the parallel processing architecture for the parallel motion-contrast pathway [42] also employing medulla inter-neurons as the core substrate. Its core regulatory mechanism is characterized by a moderate inhibitory effect of the contrast pathway on the motion pathway—a highly specific inhibition that primarily serves to attenuate interference from high-contrast motion signals. Inspired by Drosophila’s visual system, the contrast normalization and parallel motion-contrast pathways have gradually been applied to the fields of wide-field motion estimation, collision detection, and small moving object detection. The relevant literature reviews are shown in Table 2. Given the effectiveness of parallel motion-contrast pathways in eliminating false positives arising from background clutter, this study integrates such pathways into the LC11-based bio-inspired visual model.

## 3. Proposed Methods

**Figure 1:**
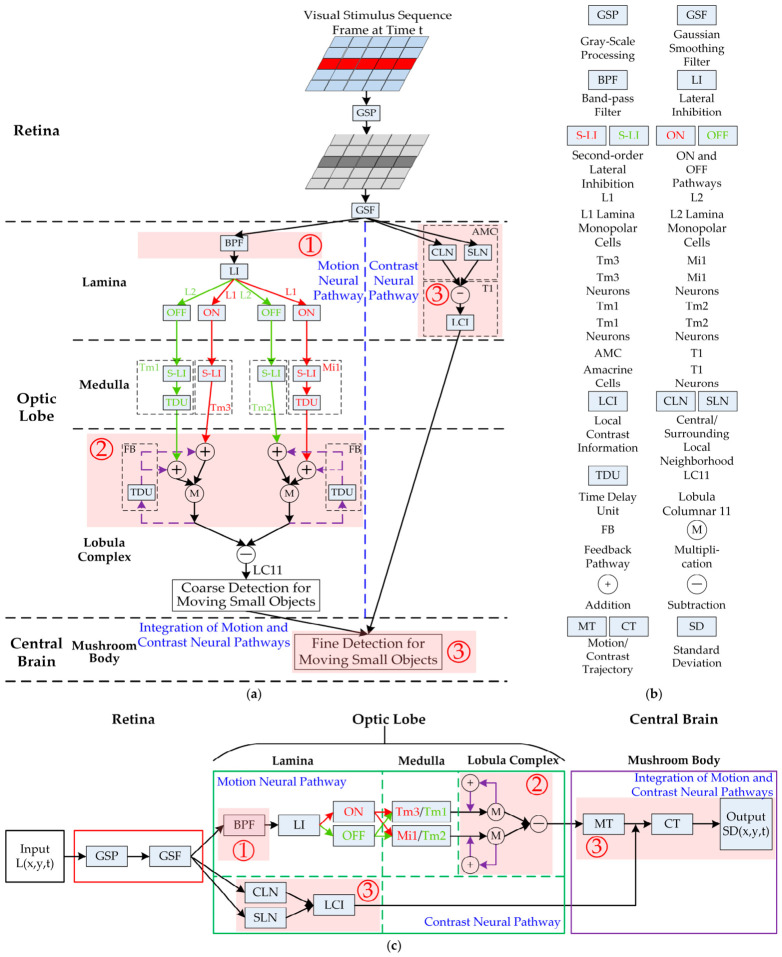
Network structure, legend and framework of the DVSOMS-MSOD model. (**a**) The network structure of the DVSOMS-MSOD model; the regions to the left and right of the blue dashed line represent the motion and contrast neural pathways, respectively; in the motion neural pathway, the red and green pipelines represent the ON and OFF motion pathways, respectively, the red and green arrows represent the transmission of the ON and OFF signals, respectively, the red and green characters represent the processing of the ON and OFF signals, respectively; the purple pipeline represents the feedback pathway, and the purple arrows represent the transmission of the feedback signal; (**b**) The legend of the DVSOMS-MSOD model; (**c**) The framework of the DVSOMS-MSOD model, and the meanings of the colors, arrows, and characters are the same as in (**a**); Meanwhile, in (**a**,**c**) the light red shadow ① represents the main contribution 1, ② represents the main contribution 2, and ③ represents the main contribution 3.

The DVSOMS-MSOD model will be developed in this section with formulation on three-order neural layers including the retina, the optic lobe, and the central brain as well as the model parameter configuration. Figure 1 depicts the network structure, legend, and framework of the DVSOMS-MSOD model. In the first-order neural layer, the visual stimuli are perceived, converted to grayscale, and blurred by R1–R6 in the retina. In the second-order neural layer, the visual signals are divided into parallel motion and contrast neural pathways. In the motion neural pathway, the visual signals are first decomposed into parallel ON and OFF neural pathway signals, then the neural feedback mechanism and the complete HRC are integrated into this pathway, finally utilizing the LC11 neuron to detect small moving objects and extract their location information. In the contrast neural pathway, the visual signals are first computed from the central and surrounding local neighborhoods, then the local contrast information is calculated. In the third-order neural layer, the mushroom body of the central brain generates motion trajectories using the location information of small moving objects, and subsequently generates contrast trajectories using the local contrast information and motion trajectories to more finely detect small moving objects.

### 3.1. Retina Neural Layer

**Figure 2:**
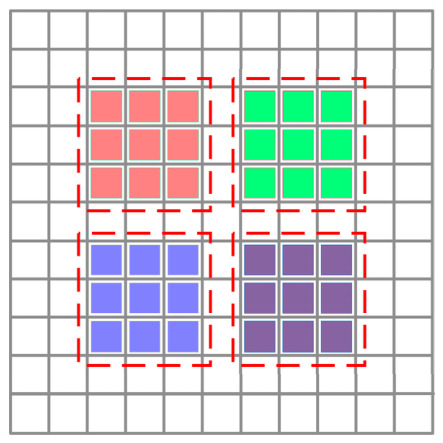
Schematic illustration of the mapping from multiple pixels to ommatidia. Among them, the red dashed rectangular areas contain multiple pixels, with each pixel corresponding to a single photoreceptor cell and each red dashed rectangular area representing the field of view of an ommatidium; the light red, light green, light blue, and light purple rectangles represent single photoreceptor cell, and the red dashed rectangles represent the ommatidia.

In the retina neural layer, Drosophila has a large number of ommatidia, each ommatidium consists of several photoreceptors, and each photoreceptor corresponds to a pixel [46]. A schematic illustration of the mapping from multiple pixels to ommatidia is shown in Figure 2. These photoreceptors are able to perceive, grayscale, and blur the visual stimuli frame-by-frame, and transmit it to downstream neurons for further processing. Specifically, this model adopts grayscale processing to obtain the grayscaled signal of luminance and the Gaussian smoothing filter to obtain the blurred signal of luminance (see GSP and GSF in the retina neural layer shown in Figure 1); these are defined in Equations (1) and (2):

(1) $$L_{\mathrm{gray}}\left(x,y,t\right)=0.2989\cdot L_{\mathrm{red}}\left(x,y,t\right)+0.5870\cdot L_{\mathrm{green}}\left(x,y,t\right)+0.1140\cdot L_{\mathrm{blue}}\left(x,y,t\right)$$

(2) $$P\left(x,y,t\right)=\iint L_{\mathrm{gray}}\left(u,v,t\right)\cdot G_{\sigma_{1}}\left(x-u,y-v\right)\mathrm{dudv}$$

where $L_{\mathrm{red}}\left(x,y,t\right)$, $L_{\mathrm{green}}\left(x,y,t\right)$, and $L_{\mathrm{blue}}\left(x,y,t\right)$ represent the luminance values of the red, green, and blue channels in the visual frame at time t; $P\left(x,y,t\right)$ represents the output response signal of the retina neural layer; $L_{\mathrm{gray}}\left(x,y,t\right)$ represents the luminance signal of each pixel in the visual frame at time t; x and y represent the horizontal and vertical coordinates of the two-dimensional (2D) visual frame; t represents the temporal coordinate of the 2D visual frame; u and v are dummy integration variables introduced to prevent variable confusion, i.e., the horizontal and vertical coordinates of the 2D visual frame; and $G_{\sigma_{1}}\left(x,y\right)$ represents the 2D Gaussian kernel, which is defined in Equation (3):

(3) $$G_{\sigma_{1}}\left(x,y\right)=\frac{1}{2{\pi \sigma }_{1}^{2}}\exp\left(-\frac{x^{2}{+y}^{2}}{2\sigma_{1}^{2}}\right)$$

where $\sigma_{1}$ represents the standard deviation of the 2D Gaussian kernel.

### 3.2. Optic Lobe Neural Layer

#### 3.2.1. Lamina Neural Sub-Layer

In the motion neural pathway, a large number of the LMC neurons (L1 and L2) exhibit high sensitivity to changes in luminance and possess the lateral inhibition mechanism, the rationality of which has been validated by relevant references (see BPF and LI in the lamina neural sub-layer of the optic lobe shown in Figure 1). Meanwhile, the signal is separated into parallel ON (the L1 type of the LMC) and OFF (the L2 type of the LMC) pathways, which encode luminance increases and decreases (see red ON and green OFF in the lamina neural sub-layer of the optic lobe shown in Figure 1), respectively.

**Figure 3:**
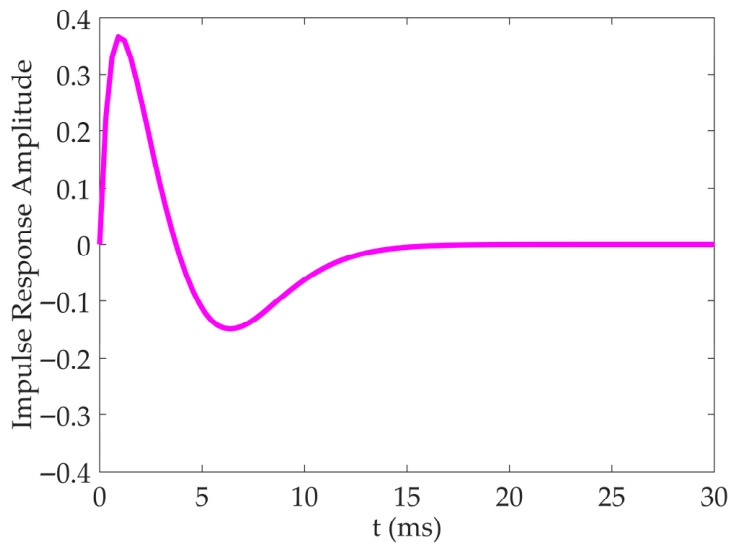
Schematic diagram of the temporal band-pass filter $H\left(t\right)$. Among them, $n_{1}$ = 1 and $n_{2}$ = 6.

In the first stage, the sensitivity of the LMC neuron to changes in luminance is modeled as the temporal band-pass filter, equivalent to the difference between consecutive frames, capable of extracting the changes in luminance. The DVSOMS-MSOD model is obtained by convolving the output of the retina neural layer with the temporal band-pass filter. Mathematically, the aforementioned convolution operation has an equivalent integral representation, which is defined in Equation (4):

(4) $$\mathrm{LMC}\left(x,y,t\right)=\int P\left(x,y,s\right)\cdot H\left(t-s\right)\mathrm{ds}$$

where $LMC\left(x,y,t\right)$ represents the response signal of the LMC neuron; s represents a dummy integration variable introduced to prevent variable confusion, i.e., representing the temporal coordinate of the visual frame; and $H\left(t\right)$ represents the improved temporal band-pass filter, which is defined in Equation (5):

(5) $$H\left(t\right)=\left(\frac{n_{2}!t^{n_{1}}-n_{1}!t^{n_{2}}}{n_{1}!n_{2}!}\right)\exp\left(-t\right)$$

where $n_{1}$ and $n_{2}$ represent two time constants, satisfying $n_{2}>n_{1}$ simultaneously. A schematic diagram of the temporal band-pass filter is shown in Figure 3.

**Figure 4:**
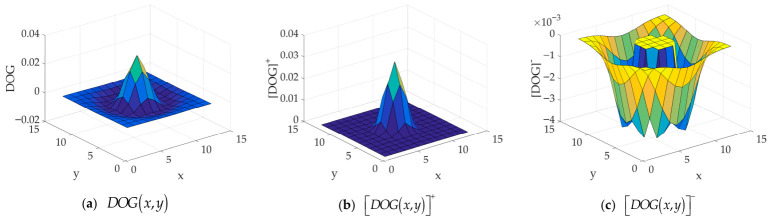
Schematic diagram of $\mathrm{DOG}\left(x,y\right)$, ${\left[\mathrm{DOG}\left(x,y\right)\right]}^{+}$, and ${\left[\mathrm{DOG}\left(x,y\right)\right]}^{-}$. Among them, $\sigma_{2}$ = 1.5 and $\sigma_{3}$ = 2.0; (**a**) represents the result of the Difference of Gaussians; (**b**) represents the maximum between the result of the Difference of Gaussians and 0; and (**c**) represents the minimum between the result of the Difference of Gaussians and 0.

In the second stage, the lateral inhibition mechanism of the LMC neuron is simulated by convolving the response signal of the LMC neuron with the inhibition kernel. Mathematically, the aforementioned convolution operation has an equivalent integral representation, which is defined in Equation (6):

(6) $${\mathrm{LMC}}_{I}\left(x,y,t\right)=∭\mathrm{LMC}\left(u,v,s\right)\cdot W_{I}\left(x-u,y-v,t-s\right)\mathrm{dudvds}$$

where ${LMC}_{I}\left(x,y,t\right)$ represents the response signal of the LMC neuron after the lateral inhibition; and $W_{I}\left(x,y,t\right)$ represents the inhibition kernel, which is defined in Equation (7):

(7) $$W_{I}\left(x,y,t\right)=W_{S}^{P}\left(x,y\right)\cdot W_{T}^{P}\left(t\right)+W_{S}^{N}\left(x,y\right)\cdot W_{T}^{N}\left(t\right)$$

where $W_{S}^{P}\left(x,y\right)$ and $W_{S}^{N}\left(x,y\right)$ represent the spatial excitatory signal and the spatial inhibitory signal, respectively; $W_{T}^{P}\left(x,y\right)$ and $W_{T}^{N}\left(x,y\right)$ represent the temporal excitatory signal and the temporal inhibitory signal, respectively, which are defined in Equations (8)–(12):

(8) $$\mathrm{DOG}\left(x,y\right)=G_{\sigma_{2}}\left(x,y\right)-G_{\sigma_{3}}\left(x,y\right)$$

(9) $$W_{S}^{P}\left(x,y\right)={\left[\mathrm{DOG}\left(x,y\right)\right]}^{+}$$

(10) $$W_{S}^{N}\left(x,y\right)={\left[\mathrm{DOG}\left(x,y\right)\right]}^{-}$$

(11) $$W_{T}^{P}\left(t\right)=\frac{1}{\lambda_{1}}\exp\left(-\frac{t}{\lambda_{1}}\right)$$

(12) $$W_{T}^{N}\left(t\right)=\frac{1}{\lambda_{2}}\exp\left(-\frac{t}{\lambda_{2}}\right)$$

where $DOG\left(x,y\right)$ represents the Difference of Gaussians, which is shown in Figure 4a; ${\left[DOG\left(x,y\right)\right]}^{+}$ represents the spatial excitatory signal ${\left[X\right]}^{+}$, corresponding to $\max\left(X,0\right)$, which is shown in Figure 4b; ${\left[DOG\left(x,y\right)\right]}^{-}$ represents the spatial inhibitory signal ${\left[X\right]}^{-}$, corresponding to $\min\left(X,0\right)$, which is shown in Figure 4c; $G_{\sigma_{2}}\left(x,y\right)$ and $G_{\sigma_{3}}\left(x,y\right)$ represent two 2D Gaussian kernels, respectively, the definitions of which are given in Equation (3); $\sigma_{2}$ and $\sigma_{3}$ represent the two standard deviations of the 2D Gaussian kernels, respectively; $\lambda_{1}$ and $\lambda_{2}$ represent two constants satisfying $\lambda_{2}>\lambda_{1}$. In the third stage, the output signal of the LMC neuron after lateral inhibition is decomposed into two neural pathway signals: luminance increase (ON) and luminance decrease (OFF), which are defined in Equation (13):

(13) $$\left\{\begin{array}{l} S_{\mathrm{ON}}\left(x,y,t\right)={\left[{\mathrm{LMC}}_{I}\left(x,y,t\right)\right]}^{+}\\ S_{\mathrm{OFF}}\left(x,y,t\right)=-{\left[{\mathrm{LMC}}_{I}\left(x,y,t\right)\right]}^{-} \end{array}\right.$$

where $S_{ON}\left(x,y,t\right)$ and $S_{OFF}\left(x,y,t\right)$ represent the ON and OFF signals, respectively.

In the contrast neural pathway, the AMC neuron directly acquires signals from the output of the ommatidia in the retina neural layer and integrates local pixel luminance signals. The AMC neuron serves two functions: on the one hand, extracting the total luminance signal within a local neighborhood centered at each spatial position; on the other hand, extracting the total luminance signal from the surrounding local neighborhood (excluding the central neighborhood); these functions are defined in Equations (14) and (15):

(14) $${\mathrm{AMC}}_{CLN}\left(x,y,t\right)=\sum_{\left(x,y\right)\in \Psi }P\left(x,y,t\right)$$

(15) $${\mathrm{AMC}}_{SLN}\left(x,y,t\right)=\left|\sum_{\left(x,y\right)\in \Pi }P\left(x,y,t\right)-\sum_{\left(x,y\right)\in \Psi }P\left(x,y,t\right)\right|$$

where $AMC_{CLN}\left(x,y,t\right)$ represents the response signal of the AMC neuron for the luminance of the central local neighborhood; $AMC_{SLN}\left(x,y,t\right)$ represents the response signal of the AMC neuron for the luminance of the surrounding local neighborhood (excluding the central local neighborhood); Ψ represents the central local neighborhood; and Π represents the surrounding local neighborhood (excluding the central local neighborhood). The T1 neuron receives the response signal of the AMC neuron and calculates the local contrast information at each position, as denoted in Equation (16):

(16) $$T1\left(x,y,t\right)=\left|\frac{1}{N_{\Pi }-N_{\Psi }}\cdot {\mathrm{AMC}}_{SLN}\left(x,y,t\right)-\frac{1}{N_{\Psi }}\cdot {\mathrm{AMC}}_{CLN}\left(x,y,t\right)\right|$$

where $T1\left(x,y,t\right)$ represents the local contrast information of the T1 neuron; $N_{\Psi }$ represents the total number of pixels in the central local neighborhood; and $N_{\Pi }$ represents the total number of pixels in the surrounding local neighborhood (excluding the central local neighborhood).

#### 3.2.2. Medulla Neural Sub-Layer

In the medulla neural sub-layer, the inter-neurons (Tm3, Tm2, Mi1, and Tm1) receive the ON and OFF signals by convolving the second-order lateral inhibition kernel (see Tm3 and Tm2 in the medulla neural sub-layer of the optic lobe shown in Figure 1). Mathematically, the aforementioned convolution operation has an equivalent integral representation, which is defined in Equation (17):

(17) $$\left\{\begin{array}{l} S_{\mathrm{Tm}3}\left(x,y,t\right)={\left[\iint S_{\mathrm{ON}}\left(u,v,t\right)\cdot W_{1}\left(x-u,y-v\right)dudv\right]}^{+}\\ S_{\mathrm{Tm}2}\left(x,y,t\right)={\left[\iint S_{\mathrm{OFF}}\left(u,v,t\right)\cdot W_{1}\left(x-u,y-v\right)dudv\right]}^{+} \end{array}\right.$$

where $S_{Tm3}\left(x,y,t\right)$ and $S_{Tm2}\left(x,y,t\right)$ represent the response signals of the Tm3 and Tm2 neurons, respectively; and $W_{1}\left(x,y\right)$ represents the second-order lateral inhibition kernel, which is defined in Equation (18):

(18) $$W_{1}\left(x,y\right)=A\cdot {\left[\mathrm{DOG}\left(x,y\right)\right]}^{+}+B\cdot {\left[\mathrm{DOG}\left(x,y\right)\right]}^{-}$$

where A and B represent two constants; and the definitions of ${\left[\mathrm{DOG}\left(x,y\right)\right]}^{+}$ and ${\left[\mathrm{DOG}\left(x,y\right)\right]}^{-}$ are given in Equations (9) and (10), respectively.

Since the responses of the Mi1 and Tm1 neurons are delayed relative to those of the Tm3 and Tm2 neurons, respectively, the DVSOMS-MSOD model employs the delayed outputs of the Tm3 and Tm2 neurons to simulate the physiological mechanisms of the Mi1 and Tm1 neurons, as denoted in Equation (19):

(19) $$\left\{\begin{array}{l} S_{\mathrm{Mi}1}\left(x,y,t\right)=\int S_{\mathrm{Tm}3}\left(x,y,s\right)\cdot \Gamma_{n_{4},\tau_{4}}\left(t-s\right)\mathrm{ds}\\ S_{\mathrm{Tm}1}\left(x,y,t\right)=\int S_{\mathrm{Tm}2}\left(x,y,s\right)\cdot \Gamma_{n_{4},\tau_{4}}\left(t-s\right)\mathrm{ds} \end{array}\right.$$

where $S_{Mi1}\left(x,y,t\right)$ and $S_{Tm1}\left(x,y,t\right)$ represent the response signals of the Mi1 and Tm1 neurons, respectively; $n_{3}$ and $\tau_{3}$ represent the order and time constant of the gamma kernel, respectively; and $\Gamma_{n_{3},\tau_{3}}\left(t\right)$ represents the gamma kernel, which is defined in Equation (20):

(20) $$\Gamma_{n,\tau }\left(t\right)={\left(\mathrm{nt}\right)}^{n}\frac{\exp\left(-\mathrm{nt}/\tau \right)}{\left(n-1\right)!\tau^{n+1}}$$

where n and τ represent the order and time constant of the gamma kernel, respectively.

#### 3.2.3. Lobula Complex Neural Sub-Layer

In the lobula complex neural sub-layer, the design of the DVSOMS-MSOD model is biologically inspired by the core functional characteristics of the LC11 neuron. The LC11 neuron integrates signals from the medulla neural sub-layer with feedback signals to selectively respond to small moving objects [47,48]. Specifically, the summation operation simulates the integration of visual signals by the LC11 neuron, while the feedback term mimics its feedback regulation mechanism that adjusts signal strength to optimize motion processing. These functional correspondences are consistent with the known physiological characteristics of the LC11 neuron. In terms of computational approximation, the feedback signal is obtained by convolving the output of the neural pathway based on the LC11 neuron with the gamma kernel [34,49], where the convolution operation allows an equivalent integral representation. The connection from medulla inter-neurons to the LC11 neuron employs the complete HRC [5,50,51]. The multiplication operation represents an engineering simplification to balance computational efficiency and biological rationality, while the feedforward regulation of the LC11 neuron involves complex electrophysiological processes that are difficult to directly simulate. But this concise computational approximation effectively realizes the core feedforward regulatory function. The complete HRC consists of two stages. In the first stage, the outputs of the two neural pathways based on the LC11 neuron are obtained by multiplying the sum of the Tm3 (Tm2) neuron output and feedback signal with the sum of the Tm1 (Mi1) neuron output and feedback signal, which are defined in Equations (21) and (22):

(21) $$\begin{array}{ll} \mathrm{LC}11_{1}\left(x,y,t\right)\;=\; & \left[S_{\mathrm{Tm}3}\left(x,y,t\right)+k\cdot \int \mathrm{LC}11_{1}\left(x,y,s\right)\Gamma_{n_{4},\tau_{4}}\left(t-s\right)\mathrm{ds}\right]\cdot \\ \; & \left[S_{\mathrm{Tm}1}\left(x,y,t\right)+k\cdot \int \mathrm{LC}11_{1}\left(x,y,s\right)\Gamma_{n_{4},\tau_{4}}\left(t-s\right)\mathrm{ds}\right] \end{array}$$

(22) $$\begin{array}{ll} \mathrm{LC}11_{2}\left(x,y,t\right)\;=\; & \left[S_{\mathrm{Tm}2}\left(x,y,t\right)+k\cdot \int \mathrm{LC}11_{2}\left(x,y,s\right)\Gamma_{n_{4},\tau_{4}}\left(t-s\right)\mathrm{ds}\right]\cdot \\ \; & \left[S_{\mathrm{Mi}1}\left(x,y,t\right)+k\cdot \int \mathrm{LC}11_{2}\left(x,y,s\right)\Gamma_{n_{4},\tau_{4}}\left(t-s\right)\mathrm{ds}\right] \end{array}$$

where $LC11_{1}\left(x,y,t\right)$ and $LC11_{2}\left(x,y,t\right)$ represent the response outputs of the two neural pathways based on the LC11 neuron, respectively; $n_{4}$ and $\tau_{4}$ represent the order and time constant of the gamma kernel, respectively; and k represents the scaling factor.

After that, the output response of the LC11 neuron is obtained by subtracting between the two neural pathways based on the LC11 neuron, as denoted in Equation (23):

(23) $$\mathrm{LC}11\left(x,y,t\right)\;=\mathrm{LC}11_{1}\left(x,y,t\right)\;-\mathrm{LC}11_{2}\left(x,y,t\right)\;$$

where $LC11\left(x,y,t\right)$ represents the response signal of the LC11 neuron.

### 3.3. Central Brain Neural Layer

In the DVSOMS-MSOD model, the mushroom body of the central brain receives response signals from the LC11 and T1 neurons [52,53]. The outputs from the LC11 and T1 neurons are integrated to distinguish small moving objects from small-object-like background features. This consists of three stages. First of all, the output of the motion neural pathway is utilized to track the position of small objects and record their motion trajectories. A threshold $T_{h}$ is set to determine the position of small objects by comparing the output of the motion neural pathway with the threshold $T_{h}$. For the threshold $T_{h}$ and a fixed time point $t_{0}$, when the output of the motion neural pathway at time point $t_{0}$ and position $\left(x_{0},y_{0}\right)$ satisfies $LC11\left(x_{0},y_{0},t_{0}\right)>T_{h}$, it can be determined that a small object is detected at position $\left(x_{0},y_{0}\right)$. Similarly, at the next time point $t_{1}$, another position $\left(x_{1},y_{1}\right)$ satisfying $LC11\left(x_{1},y_{1},t_{1}\right)>T_{h}$ can be tracked and recorded. Particularly, when the position at time point $t_{1}$ is within a small neighborhood of the position at time point $t_{0}$, it is determined that positions $\left(x_{0},y_{0}\right)$ and $\left(x_{1},y_{1}\right)$ belong to the same trajectory. By repeating the above steps, a motion trajectory can be obtained over the entire time period, as denoted in Equation (24):

(24) $$MT\;=\left(x\left(t\right),y\left(t\right)\right),\;\;\;t\in \left[t_{0},t_{n}\right]$$

where MT represents the motion trajectory; $x\left(t\right)$ and $y\left(t\right)$ represent the horizontal and vertical coordinates of the pixel as it moves over time t, respectively; $t_{0}$ and $t_{n}$ represent the initial and current times of the motion, respectively.

Then, the contrast trajectory is detected utilizing the position of each motion trajectory and the local contrast information of the T1 neuron, as denoted in Equation (25):

(25) $$CT=\left(T1(x\left(t\right)),T1\left(y\left(t\right)\right)\right),\;\;\;t\in \left[t_{0},t_{n}\right]$$

where CT represents the contrast trajectory.

Finally, the standard deviation of contrast at each time point t for each trajectory is calculated using Equation (26).

(26) $$SD\left(x,y,t\right)=\left(x\left(t\right),y\left(t\right),t,CT\right),\;\;\;t\in \left[t_{0},t_{n}\right]$$

where $\mathrm{SD}\left(x,y,t\right)$ represents the standard deviation of contrast information. Small objects are detected by performing a threshold comparison on the contrast standard deviation $\mathrm{SD}\left(x,y,t\right)$ at each time point; that is, when the contrast standard deviation of a pixel $\left(x_{0},y_{0}\right)$ at time point $t_{0}$ exceeds the threshold $T_{h2}$, the small object is determined to be detected.

In summary, the mushroom body of the central brain integrates the outputs of the LC11 and T1 neurons to suppress false positives through a three-stage process: first of all, motion trajectories are generated by thresholding LC11 responses and tracking temporally adjacent detections; second, contrast trajectories are constructed by mapping T1-generated local contrast onto each motion trajectory; finally, small moving objects are confirmed by thresholding the standard deviation of contrast along each trajectory, thereby distinguishing genuine small moving objects from small-object-like background features.

The pseudo-code of the DVSOMS-MSOD model is depicted in Algorithm 1, which shows the motion and contrast signal processing of the DVSOMS-MSOD model in detail.

**Algorithm 1.** DVSOMS-MSOD Model
  1:Input: Continuous sequence of visual stimulus frames, namely $L\left(x,y,t\right)<t=1,2,3,\ldots ,n-2,n-1,n>$.  2:Output: The detection results for the small moving object, namely $LC11\left(x,y,t\right)$ and $\mathrm{SD}\left(x,y,t\right)$.  3:Procedure DVSOMS-MSODModel_CALCULATION ($L_{t}$)  4:   for number = from ($L_{1}$) to ($L_{n}$)  5:     // 1. Retina neural layer  6:     Firstly, compute the grayscale processing of the visual stimulus sequence frames using Equation (1), get $L_{\mathrm{gray}}\left(x,y,t\right)$  7:     Then, compute the blurring mechanism of the visual stimulus sequence frames using Equations (2) and (3), get $P\left(x,y,t\right)$  8:     // 2. Optic lobe (lamina, medulla, and lobula complex) neural layer  9:     // 2.1. Lamina neural sub-layer10:      Firstly, compute the response signal of the LMC neurons using Equations (4) and (5), get $LMC\left(x,y,t\right)$11:      Secondly, compute the response signal of the LMC neuron after lateral inhibition using Equations (6)–(12), get $LMC_{I}\left(x,y,t\right)$12:      Thirdly, compute the ON and OFF signals using Equation (13), get $S_{ON/OFF}\left(x,y,t\right)$13:      Fourthly, compute the response signal of the AMC neuron using Equations (14) and (15), get $AMC_{CLN}\left(x,y,t\right)$ and $AMC_{SLN}\left(x,y,t\right)$14:      Finally, compute the response signal of the T1 neuron using Equation (16), get $T1\left(x,y,t\right)$15:      // 2.2. Medulla neural sub-layer16:      Firstly, compute the response signals of the Tm3 and Tm2 neurons using Equations (17) and (18), get $S_{Tm3/Tm2}\left(x,y,t\right)$17:      Then, compute the response signals of the Mi1 and Tm1 neurons using Equations (19) and (20), get $S_{Mi1/Tm1}\left(x,y,t\right)$18:      // 2.3. Lobula complex neural sub-layer19:      Compute the response signal of the LC11 neuron using Equations (21)–(23), get $LC11\left(x,y,t\right)$20:      // 3. Central brain (mushroom body) neural layer21:      Firstly, record the motion trajectory using Equation (24), get MT22:      Then, record the contrast trajectory using Equation (25), get CT23:      Finally, compute the standard deviation of contrast information at each time point t for each trajectory using Equation (26), and perform threshold comparison, get $\mathrm{SD}\left(x,y,t\right)$24:      return $LC11\left(x,y,t\right)$ and $\mathrm{SD}\left(x,y,t\right)$25:   end for26:End procedure


### 3.4. Model Parameter Configuration

The parameter configuration for the DVSOMS-MSOD model is given in Table 3. All parameters are determined empirically, with consideration for the functionality of the visual neural system of Drosophila.

## 4. Results

In the experiments, the DVSOMS-MSOD model was set up in Matlab R2016a (The MathWorks Corporation, Natick, MA, USA), and the hardware was an Intel Core i7-8700 running at 3.20 GHz, with 12 central processing units (CPUs). The synthetic continuous visual stimulus sequences were generated by the Vision Egg 1.1.1 [54]. The benchmark parameters of the continuous visual stimulus sequence are divided into two parts: on the one hand, the resolution and frame rate of the continuous visual stimulus sequence, which are set to 500 × 250 pixels × pixels and 30 frames per second (FPS); on the other hand, the parameter settings of the small moving object, where the luminance and size of the small moving object are set to 0 and 5 × 5 pixels × pixels, respectively, and the motion velocities of both the small moving object and the environmental background are set to 250 pixels/second. The performance of the DVSOMS-MSOD model is verified by three experiments, namely, comparative analysis of the contribution of each neural layer in the DVSOMS-MSOD model, comparative analysis of conventional machine learning methods for moving object detection, and comparative analysis of bio-inspired visual models for small moving object detection.

### 4.1. Contribution of Each Neural Layer in DVSOMS-MSOD Model

**Figure 5:**
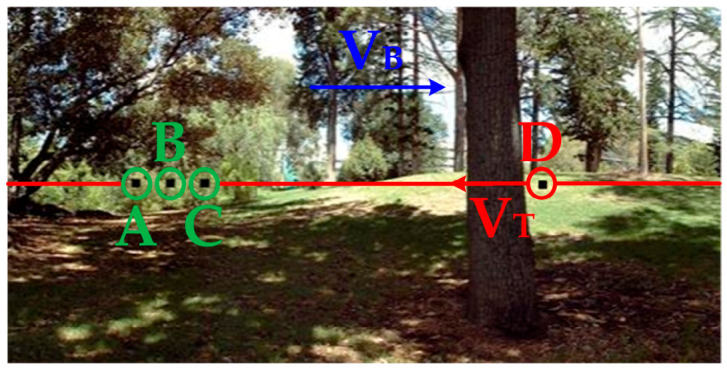
A sample frame from continuous visual stimuli sequences. The black rectangle highlighted by the red circle represents a small moving object (i.e., D); the black rectangles highlighted by the green circles represent three false small objects of the environmental background (i.e., A, B, and C); the red arrow represents the motion direction of the small object; and the blue arrow represents the motion direction of the environmental background.

**Figure 6:**
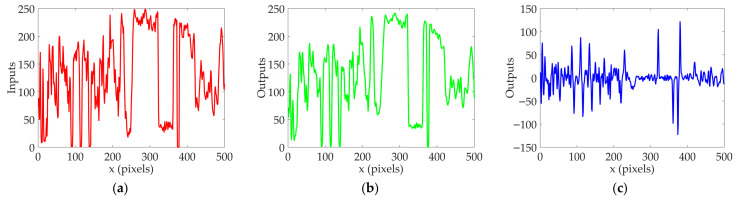

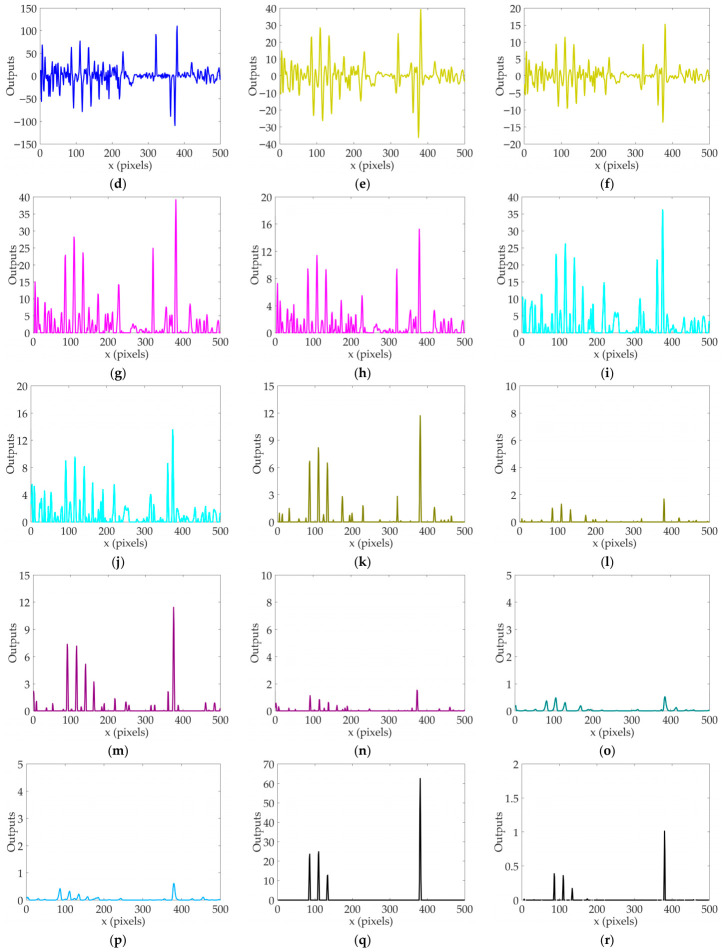
Response signals of related neurons in the retina and optic lobe neural layers of the ESTMD and DVSOMS-MSOD models. Among them, the horizontal axis represents the width of the visual stimulus sequence; and the vertical axis represents the response outputs at $t_{0}=500$ ms and $y_{0}=125$. (**a**) The input signal of the retina neural layer, the red curve represents the retinal input variation; (**b**) The output response signal of the retina neural layer, the green curve represents the retinal output variation; (**c**) The response signal of the LMC neuron of the lamina neural sub-layer in the ESTMD model, the blue curve represents the LMC output variation, same below; (**d**) The response signal of the LMC neuron of the lamina neural sub-layer in the DVSOMS-MSOD model; (**e**) The response signal of the LMC neuron of the lamina neural sub-layer after lateral inhibition processing in the ESTMD model, the dark yellow curve represents the LMC output variation after lateral inhibition processing, same below; (**f**) The response signal of the LMC neuron of the lamina neural sub-layer after lateral inhibition processing in the DVSOMS-MSOD model; (**g**) The ON signal of the lamina neural sub-layer in the ESTMD model, the magentacurve represents the ON output variation, same below; (**h**) The ON signal of the lamina neural sub-layer in the DVSOMS-MSOD model; (**i**) The OFF signal of the lamina neural sub-layer in the ESTMD model, the cyan curve represents the OFF output variation, same below; (**j**) The OFF signal of the lamina neural sub-layer in the DVSOMS-MSOD mode; (**k**) The response signal of the Tm3 neuron of the medulla neural sub-layer in the ESTMD model, the dark olive curve represents the Tm3 output variation, same below; (**l**) The response signal of the Tm3 neuron of the medulla neural sub-layer in the DVSOMS-MSOD model; (**m**) The response signal of the Tm2 neuron of the medulla neural sub-layer in the ESTMD model, the dark purple curve represents the Tm2 output variation, same below; (**n**) The response signal of the Tm2 neuron of the medulla neural sub-layer in the DVSOMS-MSOD model; (**o**) The response signal of the Tm1 neuron of the medulla neural sub-layer in the DVSOMS-MSOD model, the dark cyan curve represents the Tm1 output variation; (**p**) The response signal of the Mi1 neuron of the lobula complex neural sub-layer in the DVSOMS-MSOD model, the light blue curve represents the Mi1 output variation; (**q**) The response signal of the STMD neuron of the lobula complex neural sub-layer in the ESTMD model, the black curve represents the STMD output variation; (**r**) The response signal of the LC11 neuron of the lobula complex neural sub-layer in the DVSOMS-MSOD model, the black curve represents the LC11 output variation.

**Figure 7:**
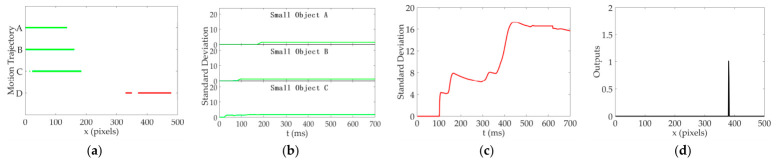
Response signals of related neurons in the central brain neural layer of the DVSOMS-MSOD model. Among them, the horizontal axis in (**a**,**d**) represents the width of the visual stimulus sequence; the horizontal axis in (**b**,**c**) represents the temporal duration of the visual stimulus sequence; and the vertical axis in (**d**) denotes the response outputs at $t_{0}=500$ ms and $y_{0}=125$. (**a**) The motion trajectories of the small objects called A, B, C, and D in the DVSOMS-MSOD model ($T_{h}$ = 0.2), the green lines denote the motion trajectories of the small objects called A, B, and C; the red line denotes the motion trajectories of the small object D; (**b**) The standard deviation of the local contrast information of the false small objects called A, B, and C in the DVSOMS-MSOD model, the green curves represent the variation of the standard deviation; (**c**) The standard deviation of the local contrast information of the small object called D in the DVSOMS-MSOD model, the red curve represents the variation of the standard deviation; (**d**) The response signal after being processed through the contrast neural pathway in the DVSOMS-MSOD model ($T_{h2}$ = 4.0), the black curve represents the DVSOMS-MSOD output variation.

To evaluate the role of each neural layer in the DVSOMS-MSOD model, it is necessary to observe and analyze the response signals of relevant neurons in each neural layer, while comparing them with the relevant neurons in each neural layer of the elementary STMD (ESTMD) model [55]. The configuration parameters can be found in the reference [22]. For the perceived visual stimulus sequence $I\left(x,y,t\right)$, the pixel size is $x\in \left[0,\;500\right]$ pixels, $y\in \left[0,\;250\right]$ pixels, with a time interval of milliseconds $t\in \left[0,\;700\right]$. In the experiments for analysis of relevant neurons in each neural layer, y was set to $y_{0}=125$ and t was set to $t_{0}=500$ milliseconds. The sample frame from continuous visual stimulus sequences is shown in Figure 5, where three pseudo-small objects, namely green circles A, B, and C, are added to the environmental background and move to the right together with the environmental background, while a small moving object, namely red circle D, is added moving to the left. The response signals of relevant neurons in the retina and optic lobe neural layers of the ESTMD and DVSOMS-MSOD models are shown in Figure 6, and the response signals of relevant neurons in the central brain neural layer of the DVSOMS-MSOD model are shown in Figure 7.

Figure 6a represents the input signal of the retina neural layer, and Figure 6b represents the output response signal of the retina neural layer. Compared with the input signal of the retina neural layer, the output response signal of the retina neural layer has undergone smoothing processing. This is because the input signal is processed through the spatial Gaussian smoothing filter in the retina neural layer, the input and output of both the ESTMD and DVSOMS-MSOD models are identical.

Figure 6c,d represent the response signal of the LMC neuron, where this output is modeled as the temporal band-pass filter of the response signal of the retina neural layer, revealing the changes in luminance at each pixel, with positive values corresponding to luminance increases and negative values corresponding to luminance decreases. The response signals of the LMC neuron in the DVSOMS-MSOD model differ from those of the ESTMD model, because the DVSOMS-MSOD model employs an improved temporal band-pass filter. Figure 6e,f represent the response signal of the LMC neuron after lateral inhibition processing. Compared with Figure 6c,d, the response signals of the LMC neuron are suppressed, because the response signals of the LMC neuron in the ESTMD and DVSOMS-MSOD models have undergone lateral inhibition processing.

Figure 6g–j represent the ON and OFF signals in the lamina neural layer of the ESTMD and DVSOMS-MSOD models, respectively. Among them, the ON signals correspond to luminance increases, while the OFF signals correspond to luminance decreases. Figure 6k–p represent the response signals of the Tm3, Tm2, Mi1, and Tm1 neurons in the medulla neural layer in the ESTMD and DVSOMS-MSOD models, respectively. The ESTMD model does not utilize the response signals of the Tm1 and Mi1 neurons. The response signals of the Tm3 and Tm2 neurons in the ESTMD and DVSOMS-MSOD models are suppressed, because both models implement the second-order lateral inhibition mechanism to achieve size selectivity.

From Figure 6q,r, it can be observed that both the ESTMD and DVSOMS-MSOD models exhibit the strongest response signal at position x = 380, indicating the location of the small moving object. However, the response signals are also observed at three positions: x = 87, 111, and 134, indicating the locations of false small objects from the environmental background. This indicates that the STMD and LC11 neurons are sensitive to small moving objects and possess size selectivity, but further optimization is required in eliminating small-object-like background features.

The results shown in Figure 7 represent the response signals of the lobula complex and mushroom body neural layers in the DVSOMS-MSOD model. Figure 7a represents the motion trajectories of the small objects called A, B, C, and D in the DVSOMS-MSOD model. Figure 7b,c represent the standard deviation of local contrast information for the objects called A, B, C, and D in the DVSOMS-MSOD model, with the horizontal axis representing frame time. Figure 7d represents the response signal of the DVSOMS-MSOD model after processing through the contrast neural pathway, demonstrating significant improvement in performance regarding the elimination of false small objects from the environmental background.

In summary, the STMD neuron in the ESTMD model and the LC11 neuron in the DVSOMS-MSOD model exhibit similarly ineffective performance in eliminating false small objects from the environmental background. However, the output response of the mushroom body in the DVSOMS-MSOD model can effectively eliminate false small objects from the environmental background. To further compare the performance of the two models, the Signal-to-Noise Ratio (SNR) and Visual Noise (VN) are employed to evaluate the output quality of the ESTMD and DVSOMS-MSOD models, with the statistical results presented in Table 4. For SNR, smaller values indicate more obvious noise, so larger SNR values are preferred; for VN, larger values indicate more obvious noise, so smaller VN values are preferred. According to the results in Table 4, the DVSOMS-MSOD model outperforms the ESTMD model in terms of environmental background suppression capability, with SNR improved by 59.86% and 25.81%, and VN reduced by 95.74% and 21.74%. Additionally, the LC11 neuron demonstrates superior environmental background noise suppression capability compared to the ESTMD model. Meanwhile, the output quality of each neural layer was recorded across both the ESTMD and DVSOMS-MSOD models. In the retina neural layer, the ESTMD and DVSOMS-MSOD models exhibited an SNR of 3.6 and a VN of 11.50. In the lamina neural sub-layer, the ON and OFF neural pathways of the ESTMD model yielded SNR/VN values of 17.2/44.25 and 10.7/43.79, respectively; conversely, the DVSOMS-MSOD model produced markedly lower SNR values of 1.9 and 7.0 for the ON and OFF pathways, with VN values of 38.30 and 38.08, respectively. In the medulla neural sub-layer, the ESTMD model reported SNR/VN metrics of 12.3/20.57 for the Tm3 neuron and 1.3/22.72 for the Tm2 neuron. The DVSOMS-MSOD model, by contrast, yielded SNR/VN values of 7.0/6.15 for the Tm3 neuron, 5.8/6.10 for the Tm2 neuron, 8.7/2.96 for the Tm1 neuron, and 8.2/3.21 for the Mi1 neuron.

To quantitatively compare the detection performance of bio-inspired visual models for small moving object detection, the detection accuracy is employed for evaluation, as defined in Equation (27):

(27) $$D_{R}=\frac{\mathrm{The}\;\mathrm{number}\;\mathrm{of}\;\mathrm{accurate}\;\mathrm{detections}}{\mathrm{The}\;\mathrm{number}\;\mathrm{of}\;\mathrm{sequence}\;\mathrm{frames}}$$

where $D_{R}$ denotes the detection accuracy. If the pixel distance between the detection result and the ground truth is within 5 pixels, the detection result is considered accurate. The values of $D_{R}$ for the ESTMD, LC11, and DVSOMS-MSOD models are 60.43%, 71.71%, and 78.29%, respectively.

### 4.2. Comparative Analysis of Conventional Machine Learning Methods for Moving Object Detection

**Figure 8:**
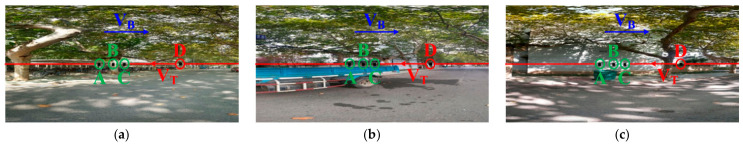

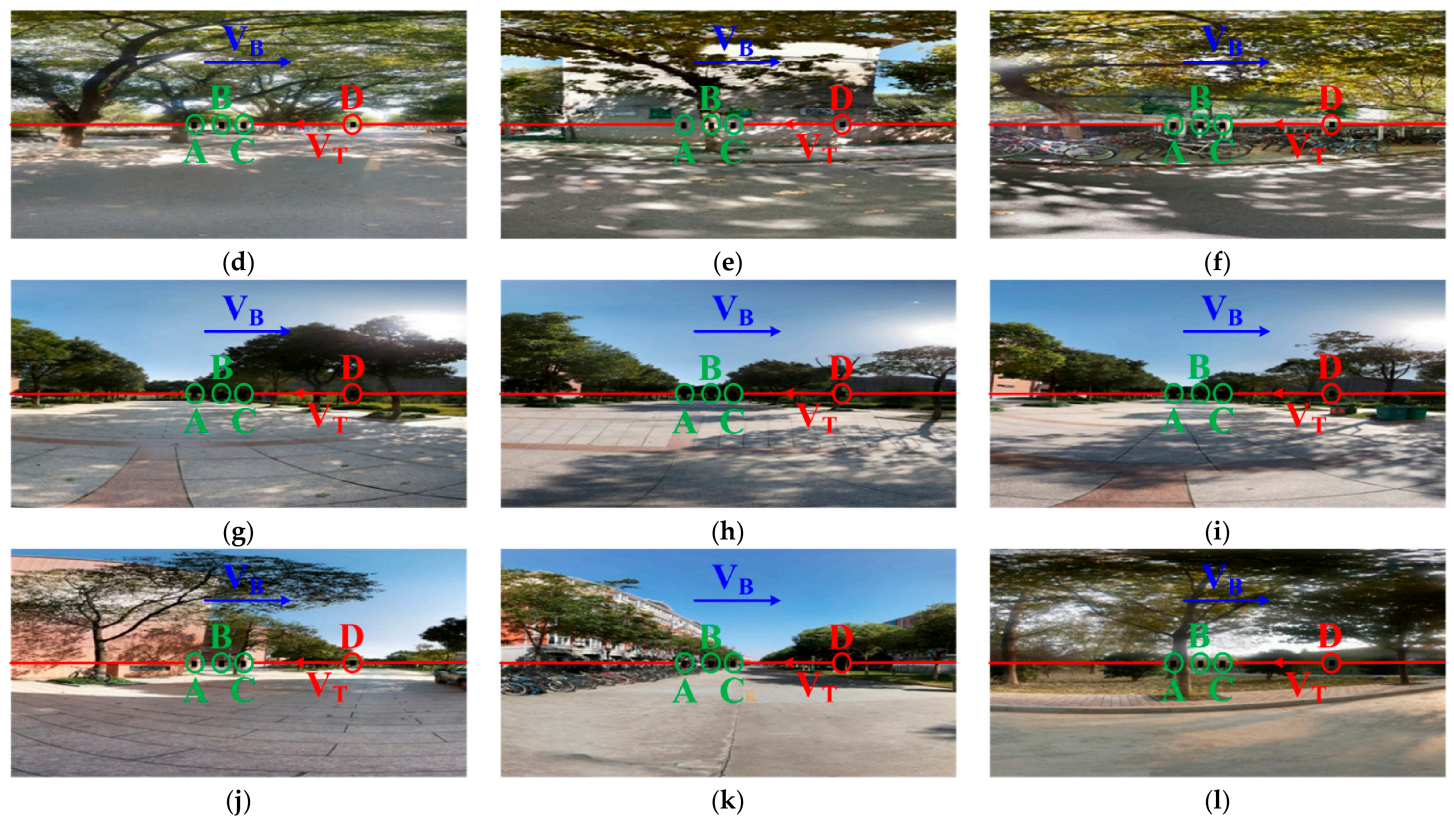
Sample images from 12 groups of continuous visual stimulus sequences. The black rectangle highlighted by the red circle represents a small moving object (i.e., D); the black rectangles highlighted by the green circles represent three false small objects of the environmental background (i.e., A, B, and C); the red arrow represents the motion direction of the small object; and the blue arrow represents the motion direction of the environmental background. (**a**–**l**) denote real-world complex environmental backgrounds 1–12, respectively.

In this section, 12 panoramic images based on real campus scenes are selected. Sample images from the 12 groups of continuous visual stimulation sequences are shown in Figure 8. The panoramic images serve as real-world complex environmental backgrounds, into which small moving objects are embedded. The time interval of the continuous visual stimulation sequences is in $t\in \left[0,\;700\right]$ milliseconds, and other parameters are set to baseline values. The black rectangles highlighted by green circles A, B, and C represent three environmental background pseudo-small objects, the black rectangle highlighted by red circle D represents a small moving object, the red arrow indicates the motion direction of the small moving object, and the blue arrow indicates the motion direction of the environmental background.

To verify the detection performance of the DVSOMS-MSOD model, it is necessary to conduct a comparative analysis with conventional moving object detection methods, including mixture of Gaussians—background subtraction model (MOG-BSM) [56], mixture of Gaussians 2—background subtraction model (MOG2-BSM) [57], Codebook [58], K-nearest neighbor—background subtraction model (KNN-BSM) [59], and visual background extractor (ViBe) [60]. Three performance indicators are employed for quantitative comparison, namely Precision (Pr), Recall (Rc), and F1-Measure (F1), which are defined in Equations (28)–(30):

(28) $$\mathrm{Pr}=\frac{\mathrm{TP}}{\mathrm{TP}+\mathrm{FP}}$$

(29) $$\mathrm{Rc}=\frac{\mathrm{TP}}{\mathrm{TP}+\mathrm{FN}}$$

(30) $$F1=2\cdot \frac{\mathrm{Pr}\cdot Rc}{\mathrm{Pr}+Rc}$$

where TP denotes the number of pixels that belong to the object category in both the detection result and the ground truth annotation; FN denotes the number of pixels that belong to the object category in the ground truth annotation but are incorrectly identified as background in the detection result; FP denotes the number of pixels that belong to the background in the ground truth annotation but are incorrectly identified as the object category in the detection result; TN denotes the number of pixels that belong to the background in both the detection result and the ground truth annotation; Pr denotes precision; Rc denotes recall; F1 denotes F1-Measure. For Pr, Rc, and F1, larger values indicate better model performance. The statistical results of the quantitative comparison of MOG-BSM, MOG2-BSM, Codebook, KNN-BSM, ViBe, and DVSOMS-MSOD are shown in Table 5.

Based on the results in Table 5, the following six conclusions can be summarized: (1) The DVSOMS-MSOD model achieved relative optimal results across all three performance metrics, namely Pr, Rc, and F1. (2) In terms of Pr, the DVSOMS-MSOD model improved by 98.37%, 98.52%, 95.41%, 95.70%, and 89.93%, respectively, with an average improvement of 95.59%. (3) In terms of Rc, the DVSOMS-MSOD model improved by 43.78%, 47.97%, 43.11%, 40.67%, and 37.95%, respectively, with an average improvement of 42.70%. (4) In terms of F1, the DVSOMS-MSOD model improved by 98.21%, 98.37%, 94.96%, 95.28%, and 89.10%, respectively, with an average improvement of 95.18%. (5) The overall detection performance of the DVSOMS-MSOD model improved by 77.82%. To compare the output quality between conventional moving object detection methods and the DVSOMS-MSOD model, SNR and VN were employed to compare the output quality of MOG-BSM, MOG2-BSM, Codebook, KNN-BSM, ViBe, and DVSOMS-MSOD, and the statistical results are shown in Table 6.

According to the results in Table 6, the DVSOMS-MSOD model achieved the relative optimal results in both SNR and VN. In terms of VN, the output quality of the DVSOMS-MSOD model outperformed that of the five conventional moving object detection methods, with improvements of 85.33%, 87.54%, 70.03%, 38.62%, and 18.14%, respectively, averaging 59.93%. In terms of SNR, the output quality of the DVSOMS-MSOD model outperformed that of the five conventional moving object detection methods, with decreases of 98.35%, 98.15%, 96.93%, 97.27%, and 96.59%, respectively, averaging 97.46%. The DVSOMS-MSOD model surpasses the five conventional moving object detection methods in environmental background suppression capability, with an overall output quality improvement of 78.70%. To compare the real-time performance between conventional moving object detection methods and the DVSOMS-MSOD model, the running time for processing every two sequential frames in real-world complex dynamic environments was compared among MOG-BSM, MOG2-BSM, Codebook, KNN-BSM, ViBe, and DVSOMS-MSOD, and the statistical results are shown in Table 7.

According to the results in Table 7, the DVSOMS-MSOD model achieved the third-best relative optimal result in terms of running time. Its real-time performance is superior to that of MOG2-BSM, KNN-BSM, and ViBe, but inferior to that of MOG-BSM and Codebook, with changes of −0.06%, 16.22%, −4.69%, 17.79%, and 23.76%, respectively, averaging a decrease of 10.60%.

### 4.3. Comparative Analysis of Bio-Inspired Visual Models for Small Moving Object Detection

To further verify the detection performance of the DVSOMS-MSOD model, the ESTMD [22,55] model and Drosophila vision neural pathway model (DVNPM) [5] are selected as baseline comparison models. The configuration parameters for the DVNPM model can be found in the reference [50]. To quantitatively compare the detection performance of bio-inspired visual models for small moving object detection, the detection accuracy defined in Equation (27) is employed for evaluation.

**Figure 9:**
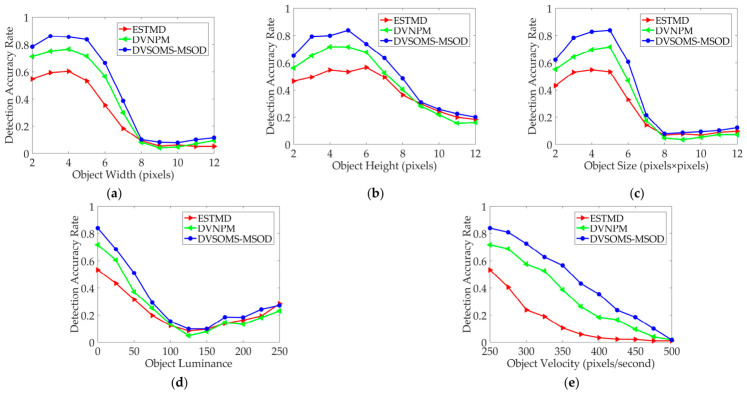
The detection accuracy rates of the ESTMD, DVNPM and DVSOMS-MSOD models in different object widths, heights, sizes, luminance, and motion velocities. (**a**) Different object widths; (**b**) Different object heights; (**c**) Different object sizes; (**d**) Different object luminance levels; (**e**) Different object motion velocities.

To evaluate the detection performance for small moving objects under varying parameters, the parameter settings are listed in Table 8. The baseline parameters are as follows: width (5 pixels), height (5 pixels), size (5 × 5 pixels × pixels), luminance (0), and motion velocity (250 pixels/second). $x_{\mathrm{start}}:x_{\mathrm{interval}}:x_{\mathrm{end}}$ denotes the initial value, interval value, and final value of the value range x. The detection accuracy rates of the ESTMD, DVNPM and DVSOMS-MSOD models in different object widths, heights, sizes, luminance, and motion velocities are shown in Figure 9.

According to the results in Figure 9a, for different object widths with opposite motion directions between the object and environmental background, the detection performance of the DVSOMS-MSOD model is superior to that of the ESTMD and DVNPM models. Specifically, first of all, when the object width ranges from 2 to 8 pixels, the detection performance of the DVSOMS-MSOD model surpasses that of both the ESTMD and DVNPM models; then, when the object width ranges from 9 to 10 pixels, the detection performance of the DVSOMS-MSOD model is slightly better than that of the ESTMD and DVNPM models, while the detection performance of the ESTMD model is slightly superior to that of the DVNPM model; finally, when the object width ranges from 11 to 12 pixels, the detection performance of the DVSOMS-MSOD model surpasses that of both the ESTMD and DVNPM models, while the detection performance of the DVNPM model is slightly superior to that of the ESTMD model.

According to the results in Figure 9b, for different object heights with opposite motion directions between the object and environmental background, the detection performance of the DVSOMS-MSOD model is superior to that of the ESTMD and DVNPM models. Specifically, first, when the object height ranges from 2 to 8 pixels, the detection performance of the DVSOMS-MSOD model surpasses that of both the ESTMD and DVNPM models; however, when the object height exceeds 8 pixels, the detection performance of the DVSOMS-MSOD model is slightly better than that of the ESTMD and DVNPM models, while the detection performance of the ESTMD model is slightly superior to that of the DVNPM model.

According to the results in Figure 9c, for different object sizes with opposite motion directions between the object and environmental background, the detection performance of the DVSOMS-MSOD model is superior to that of the ESTMD and DVNPM models. Specifically, when the object size ranges from 2 × 2 to 7 × 7 pixels × pixels, the detection performance of the DVSOMS-MSOD model surpasses that of both the ESTMD and DVNPM models; however, when the object size exceeds 8 × 8 pixels × pixels, the detection performance of the DVSOMS-MSOD model is comparable to that of the ESTMD and DVNPM models.

According to the results in Figure 9d, for different object luminance levels with opposite motion directions between the object and environmental background, the detection performance of the DVSOMS-MSOD model is superior to that of the ESTMD and DVNPM models. Specifically, when the object luminance ranges from 0 to 75, the detection advantage of the DVSOMS-MSOD model is superior to that of the ESTMD and DVNPM models; however, when the object luminance exceeds 100, the detection performances of the ESTMD, DVNPM, and DVSOMS-MSOD models are comparable.

According to the results in Figure 9e, for different object motion velocities with opposite motion directions between the object and environmental background, the detection performance of the DVSOMS-MSOD model is superior to that of the ESTMD and DVNPM models. Specifically, when the object motion speed ranges from 250 to 475 pixels/second, the detection performance of the DVSOMS-MSOD model surpasses that of both the ESTMD and DVNPM models; however, when the object motion speed reaches 500 pixels/second, the detection performances of the ESTMD, DVNPM, and DVSOMS-MSOD models are comparable and approach zero.

In summary, compared with the ESTMD model, the detection accuracy of the DVSOMS-MSOD model improved by 35.80%, 26.09%, 33.53%, 28.00%, and 66.73%, respectively, with an average improvement of 38.03%; compared with the DVNPM model, the detection accuracy of the DVSOMS-MSOD model improved by 14.81%, 14.50%, 19.24%, 18.57%, and 25.15%, respectively, with an average improvement of 18.45%. Therefore, the DVSOMS-MSOD model outperforms both the ESTMD and DVNPM models in terms of detection accuracy, with an overall improvement of 28.24%. Meanwhile, the Pr, Rc, and F1 performance indicators are employed to quantitatively compare the ESTMD, DVNPM, and DVSOMS-MSOD models, and the statistical results are shown in Table 9.

According to the results in Table 9, the following six conclusions can be summarized: (1) The DVSOMS-MSOD model achieved the relative optimal results in Pr, Rc, and F1. (2) In terms of Pr, the DVSOMS-MSOD model improved by 58.22% and 33.19%, respectively, with an average improvement of 45.71%. (3) In terms of Rc, the DVSOMS-MSOD model improved by 47.66% and 29.52%, respectively, with an average improvement of 38.59%. (4) In terms of F1, the DVSOMS-MSOD model improved by 57.45% and 32.87%, respectively, with an average improvement of 45.16%. (5) The detection performance of the DVSOMS-MSOD model improved by 43.15%. To compare the real-time performance of the ESTMD, DVNPM, and DVSOMS-MSOD models, their running times for processing every two sequential frames in real-world complex dynamic environments were compared, and the statistical results are shown in Table 10.

According to the results in Table 10, the DVSOMS-MSOD model is inferior to the ESTMD and DVNPM models in terms of real-time performance, with increases of 53.62% and 33.17%, respectively, averaging 43.40%. Compared with the ESTMD and DVNPM models, the DVSOMS-MSOD model incorporates three additional modules: neural feedback, contrast neural pathway, and integration of motion and contrast neural pathways, resulting in inferior real-time performance compared to the ESTMD and DVNPM models.

## 5. Discussion

The robust detection of small moving objects in real-world complex dynamic environments represents a formidable challenge in computer vision. The LC11 neuron within the Drosophila visual neural system demonstrates remarkable selectivity for small moving objects, providing an exceptional biological paradigm for addressing this computational challenge. Despite extensive neurophysiological characterization of the LC11 neuron, corresponding computational modeling efforts remain comparatively underdeveloped.

Through systematic analysis of the neural circuitry encompassing the LC11 neuron and mushroom body, this study delineates a canonical visual processing hierarchy: photoreceptor signals undergo initial transduction in the retinal layer, subsequent parallel processing via motion and contrast pathways in the lamina layer, distributed processing across specialized visual inter-neurons, and integrative convergence by the mushroom body. Grounded in this neuroarchitectural framework, we present the DVSOMS-MSOD model—a three-layer computational architecture that exploits the exquisite sensitivity of the LC11 neuron and mushroom body to enable precise detection of small moving objects. The model incorporates several biologically motivated enhancements: an optimized temporal band-pass filter, recurrent neural feedback mechanisms for signal amplification, and complete HRC for elementary motion computation [5,50]. Critically, through the functional integration of motion and contrast pathways within the mushroom body computational module, the model achieves effective suppression of background-induced pseudo-small objects, thereby substantially enhancing detection fidelity in real-world complex dynamic environments [26,45].

Rigorous validation of the DVSOMS-MSOD model was conducted through three complementary experimental approaches. First, layer-wise activation analysis was performed to elucidate the hierarchical computational mechanisms underlying progressive motion feature extraction and object detection. Second, comprehensive benchmarking against 12 diverse datasets was executed, comparing model performance against both conventional machine learning methods and contemporary bio-inspired detection models. Experimental results substantiate the efficacy of the DVSOMS-MSOD model in small moving object detection, with particular advantages manifested in challenging real dynamic contexts characterized by complex backgrounds and variable illumination.

Acknowledgment of model limitations is essential for scientific transparency. The current architecture exhibits two primary constraints: (1) functional specificity for small moving objects precludes generalization to normal-sized moving objects, thereby circumscribing the operational domain; (2) the postulated contrast pathway, while computationally efficacious, awaits definitive neuroanatomical corroboration and should be regarded as a working hypothesis rather than established biological fact; the existence of alternative neural mechanisms for small-object-like background feature suppression in the Drosophila visual neural system remains an open question meriting continued investigation; and (3) the evaluation in Section 4.2 and Section 4.3 employs a synthetic video dataset generated by embedding virtual small moving objects into real-world panoramic backgrounds. While this approach enables precise manipulation of object attributes (e.g., scale, velocity, trajectory) and ensures experimental reproducibility, it suffers from several limitations. First, virtual objects may exhibit unrealistic texture details and lighting interactions, potentially leading to over-optimistic performance estimates. Second, the motion patterns of virtual objects fail to capture the stochastic behaviors of real-world entities such as pedestrians and vehicles. Furthermore, the real panoramic backgrounds remain static, overlooking dynamic environmental factors, including illumination variations and weather effects. These limitations collectively constrain the validation of the model’s applicability in practical scenarios. To address these issues, future work will be dedicated to constructing large-scale fully real-world panoramic datasets with fine-grained annotations, conducting systematic bias analyses to quantify the domain shift between virtual and real objects, and exploring domain adaptation and randomization techniques to bridge this gap, thereby enhancing the model’s real-world generalization capability.

In summary, this investigation advances our understanding the function of the LC11 neuron in visual motion processing through quantitative characterization of its response dynamics to small moving objects. By demonstrating the computational utility of contrast pathway integration for suppressing small-object-like background features, we establish the feasibility of Drosophila-inspired neural architectures for robust object detection in real-world complex dynamic environments. Systematic experimental validation substantiates the biological plausibility and engineering utility of these mechanisms, while critical discussion of model limitations provides a principled foundation for subsequent theoretical and experimental elaboration.

## 6. Conclusions

In this study, we present the DVSOMS-MSOD model, which leverages the LC11 neuron and mushroom body of Drosophila to provide a biologically principled solution for small moving object detection in real-world complex dynamic environments. Compared with conventional machine learning methods and existing bio-inspired visual models, the proposed DVSOMS-MSOD model achieves notable improvements in detection performance, output quality, and accuracy while maintaining favorable runtime efficiency. These gains stem from two synergistic neural computations: (1) LC11-mediated motion sensitivity, enabled by the complete HRC and medulla–lobula complex feedback, for detecting small moving objects; and (2) mushroom body-driven integration of motion trajectories with local contrast information to suppress background clutter and eliminate false positives. Although neuroanatomical evidence supporting the mushroom body’s integration of motion and contrast pathways remains to be fully established, the effectiveness of the contrast pathway in suppressing background interference and eliminating spurious small objects has been empirically validated. The relatively elevated computational cost compared with other bio-inspired visual models reflects the biological fidelity of parallel ON and OFF processing and feedback mechanisms. This research broadens the dimensional scope of investigations into the visual neural mechanism of Drosophila, offering novel theoretical frameworks and technical pathways for the interdisciplinary convergence of neuromorphic intelligence, computer vision, and neuroscience.

## Figures and Tables

**Table 1:** Literature review concerning bio-inspired models with neural feedback mechanisms.

Research Field	References	Core Principle	Main Contributions	Limitations
Small Moving Object Detection	Wang et al. [34] proposed a feedback STMD model.	Feeds the output signal back to the medulla after a temporal delay.	Effectively suppresses interference from slowly moving backgrounds while maintaining sensitivity to small fast-moving objects.	The detection performance for small moving objects under fast-moving backgrounds is unsatisfactory.
	Ling et al. [35] proposed a new feedback STMD model.	Feeds the output signal back to the lamina after a temporal delay.	Maintains a minor effect to fast-moving objects, while significantly suppressing those with lower velocities.	When the velocity of the moving object is lower than that of the background, its detection performance needs further improvement.
Collision Detection	Chang et al. [36] proposed a feedback LGMD model.	Feeds back the output signals of the ON and OFF neural pathways to their initial neurons.	Realizes compatibly the functional characteristics of both LGMD1 and LGMD2, enabling the recognition of different collision scenarios.	Sensitive to surface luminance variations of looming objects.
	Chang et al. [37] proposed a new feedback LGMD model with the ON neural pathway.	Integrates feedforward networks with the ON contrast feedback pathway.	Maintains robust selectivity for surface luminance variations of looming objects and implements diverse selectivity for ON-contrast and OFF-contrast motion.	Easily affected by visual fluctuations caused by jittery flow inputs.
	Chang et al. [38] proposed a novel feedback LGMD model with dynamic temporal variance.	Extracts dynamic temporal information from the medulla output, evaluates and adaptively regulates fluctuations in local neural responses, and feeds the corrected signal back to the retina via a negative feedback loop.	Significantly reduces the impact of fluctuating signals caused by jittery flow inputs.	The detection performance is unsatisfactory under conditions of discontinuous motion and abrupt, strong light changes.

**Table 2:** Literature review concerning bio-inspired models with contrast normalization and parallel motion-contrast pathways.

Research Field	References	Core Principle	Main Contributions	Limitations
Wide-Field Motion Estimation	Fu et al. [28] proposed an LPTC model with parallel motion-contrast pathways.	Integrates contrast normalization and motion-contrast parallel pathways into the medulla and lobula complex.	Demonstrates robustness in estimating the motion direction of wide-field objects in natural scenarios with significant input variations.	The selection of the baseline sensitivity in contrast normalization has a significant impact on model performance.
Collision Detection	Hua & Liu et al. [43,44] proposed an LPLC2 model with parallel motion-contrast pathways.	Integrates contrast normalization and motion-contrast parallel pathways into the medulla and lobula complex.	Enhances robustness of collision detection of looming objects in natural scenarios with significant input variations.	Only detects expanding motion from the center of the receptive field and remains nearly nonresponsive to looming objects starting from the periphery or corners.
Small Moving Object Detection	Wang & Ling et al. [26,45] proposed an STMD model with parallel motion-contrast pathways.	Integrates motion pathway (spatio-temporal features) and contrast pathway (local contrast features) from retina to lobula complex.	Distinguishes real small moving objects from interference and reduces false positive rates.	When the luminance levels of the small object and the background are comparable, the detection capability degrades significantly.

**Table 3:** Parameter configuration for the DVSOMS-MSOD model.

Parameters	Description	Value
$\sigma_{1}$	The standard deviation in Equations (2) and (3)	1
$n_{1}$, $n_{2}$	The time constants in Equation (5)	1, 6
$\sigma_{2}$, $\sigma_{3}$	The standard deviations in Equation (8)	1.5, 2.0
$\lambda_{1}$, $\lambda_{2}$	The constants in Equations (11) and (12)	3, 9
Ψ	The central local neighborhood in Equations (14)–(16)	11 × 11
Π	The surrounding local neighborhood in Equations (15) and (16)	31 × 31
A, B	The constants in Equation (18)	1, 3
$n_{3}$, $\tau_{3}$	The order and time constant in Equation (19)	5, 25
$n_{4}$, $\tau_{4}$	The order and time constant in Equations (21) and (22)	10, 25
k	The scaling factor in Equations (21) and (22)	0.01

**Table 4:** Quantitative comparison of statistical results for the output quality in the ESTMD, LC11, and DVSOMS-MSOD models.

Indicators	ESTMD	LC11	DVSOMS-MSOD
*SNR*	11.2	20.7	**27.9**
*VN*	29.56	1.61	**1.26**

Note: The bold data indicates the relative optimal result.

**Table 5:** Quantitative comparison statistical results for the performance indicators in the MOG-BSM, MOG2-BSM, Codebook, KNN-BSM, and ViBe methods and the DVSOMS-MSOD model.

Indicators	MOG- BSM	MOG2- BSM	Codebook	KNN- BSM	ViBe	DVSOMS- MSOD
*Pr*	0.0011	0.0010	0.0031	0.0029	0.0068	0.0675
*Rc*	0.3852	0.3565	0.3898	0.4065	0.4252	0.6852
F1	0.0022	0.0020	0.0062	0.0058	0.0134	0.1229

Note: The red, green, and blue represent the first, second, and third relative optimal results, respectively.

**Table 6:** Quantitative comparison statistical results for output quality in the MOG-BSM, MOG2-BSM, Codebook, KNN-BSM, and ViBe methods and the DVSOMS-MSOD model.

Indicators	MOG- BSM	MOG2- BSM	Codebook	KNN- BSM	ViBe	DVSOMS- MSOD
*SNR*	4.19	3.56	8.56	17.53	23.38	28.56
*VN*	78.56	70.33	42.38	47.59	38.16	1.30

Note: The red, green, and blue represent the first, second, and third relative optimal results, respectively.

**Table 7:** Quantitative comparison statistical results of the running time for each sequence frame of the real-world complex dynamic environment in the MOG-BSM, MOG2-BSM, Codebook, KNN-BSM, and ViBe methods and the DVSOMS-MSOD model.

Indicators	MOG- BSM	MOG2- BSM	Codebook	KNN- BSM	ViBe	DVSOMS- MSOD
Running Time (milliseconds)	103.1258	98.5612	98.5612	125.5146	135.3516	103.1859

Note: The red, green, and blue represent the first, second, and third relative optimal results, respectively.

**Table 8:** Setting parameters of the small moving object. The experiments consist of five groups: the first group varies the width of the small moving object, the second group varies the height of the small moving object, the third group varies both the width and height of the small moving object simultaneously, the fourth group varies the luminance of the small moving object, and the fifth group varies the motion velocity of the small moving object.

	Parameters of Small Moving Object	Width (Pixels)	Height (Pixels)	Size (Pixels × Pixels)	Luminance	Motion Velocity (Pixels/Second)
Experimental Group						
Experiment 1		2:1:12	5	(2:1:12) × 5	0	250
Experiment 2		5	2:1:12	5 × (2:1:12)	0	250
Experiment 3		2:1:12	2:1:12	(2:1:12) × (2:1:12)	0	250
Experiment 4		5	5	5 × 5	0:25:250	250
Experiment 5		5	5	5 × 5	0	250:50:500

**Table 9:** Quantitative comparison statistical results for the performance indicators in the ESTMD, DVNPM, and DVSOMS-MSOD models.

Indicators	ESTMD	DVNPM	DVSOMS -MSOD
*Pr*	0.0282	0.0451	**0.0675**
*Rc*	0.3586	0.4829	**0.6852**
F1	0.0523	0.0825	**0.1229**

Note: The bold data indicates the relative optimal result.

**Table 10:** Quantitative comparison statistical results of the running time for each sequence frame of the real-world complex dynamic environment in the ESTMD, DVNPM, and DVSOMS-MSOD models.

Indicators	ESTMD	DVNPM	DVSOMS -MSOD
Running Time (milliseconds)	**47.8596**	68.9565	103.1859

Note: The bold data indicates the relative optimal result.

## Data Availability

The original contributions presented in this study are included in the article. Further inquiries can be directed to the corresponding author.
